# CD98 defines a metabolically flexible, proinflammatory subset of low‐density neutrophils in systemic lupus erythematosus

**DOI:** 10.1002/ctm2.1150

**Published:** 2023-01-18

**Authors:** Katherine R. Martin, Jessica A. Day, Jacinta A. Hansen, Damian B. D'Silva, Huon L. Wong, Alexandra Garnham, Jarrod J. Sandow, Brunda Nijagal, Nicholas Wilson, Ian P. Wicks

**Affiliations:** ^1^ Walter and Eliza Hall Institute of Medical Research Parkville Victoria Australia; ^2^ Department of Medical Biology University of Melbourne Parkville Victoria Australia; ^3^ Department of Rheumatology Royal Melbourne Hospital Parkville Victoria Australia; ^4^ Metabolomics Australia Bio21 Institute of Molecular Science and Biotechnology University of Melbourne Parkville Victoria Australia; ^5^ CSL Parkville Victoria Australia

**Keywords:** CD98, granulocyte colony‐stimulating factor, low‐density neutrophils, systemic lupus erythematosus

## Abstract

**Background:**

Low‐density neutrophils (LDN) are a distinct subset of neutrophils rarely detected in healthy people but appear in the blood of patients with autoimmune diseases, including systemic lupus erythematosus (SLE), and are mobilised in response to granulocyte colony‐stimulating factor (G‐CSF). The aim of this study was to identify novel mechanisms responsible for the pathogenic capacity of LDN in SLE.

**Methods:**

Neutrophils were isolated from donors treated with G‐CSF, and whole‐cell proteomic analysis was performed on LDN and normal‐density neutrophils.

**Results:**

CD98 is significantly upregulated in LDN from G‐CSF donors and defines a subset of LDN within the blood of SLE patients. CD98 is a transmembrane protein that dimerises with L‐type amino acid transporters. We show that CD98 is responsible for the increased bioenergetic capacity of LDN. CD98 on LDN mediates the uptake of essential amino acids that are used by mitochondria to produce adenosine triphosphate, especially in the absence of glucose. Inhibition of CD98 reduces the metabolic flexibility of this population, which may limit their pathogenic capacity. CD98^+^ LDN produce more proinflammatory cytokines and chemokines than their normal density counterparts and are resistant to apoptosis, which may also contribute to tissue inflammation and end organ damage in SLE.

**Conclusions:**

CD98 provides a phenotypic marker for LDN that facilitates identification of this population without density‐gradient separation and represents a novel therapeutic target to limit its pathogenic capacity.

## Graphical abstract

1. LDN are a longer lived proinflammatory subset of neutrophils mobilised to peripheral blood in response to G‐CSF and in SLE.
2. LDN are proteomically and metabolically distinct from normal neutrophils.
3. CD98 provides a phenotypic marker to identify LDN without the need for a density gradient and may contribute to the pathogenic capacity of this subset.

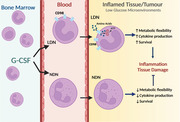

## 1 BACKGROUND

Neutrophils are the most abundant leukocytes in human peripheral blood (PB) and can migrate rapidly to extravascular sites of tissue damage, inflammation or infection. Within tissues, neutrophils provide first‐line anti‐microbial defence via degranulation, phagocytosis, generation of reactive oxygen species (ROS) and neutrophil extracellular traps (NETs).^1^, ^2^ While long considered to be a homogeneous population, it is now clear that PB neutrophils vary in maturation, phenotype and function and exert either proinflammatory or immunosuppressive effects, depending on the physiological and pathological context.^3^, ^4^

Low‐density neutrophils (LDN) are a subset of neutrophils that co‐purify with mononuclear cells during density‐gradient separation of PB. LDN are rarely detected in healthy individuals, but this population expands in patients with autoimmune disease,^5^, ^6^, ^7^ systemic inflammation,^8^ infection^9^ and cancer.^10^ LDN are also mobilised to PB following administration of granulocyte colony‐stimulating factor (G‐CSF) to healthy individuals for allogenic stem cell transplantation.^11^ Increasing evidence suggests that LDN play a role in numerous diseases, but why this occurs remains poorly understood. In the context of systemic lupus erythematosus (SLE), LDN have been shown to undergo NETosis more readily than normal‐density neutrophils (NDN), which could promote inflammation and endothelial cell damage.^6^, ^12^ LDN from SLE patients can activate T cells and induce the production of proinflammatory cytokines including interferon (IFN)γ and tumor necrosis factor (TNF).^13^ LDN counts in SLE are strongly associated with disease activity,^13^, ^14^ but the application of LDN as a potential biomarker in SLE and other clinical settings has been limited by the lack of cell surface markers and the need for density‐gradient separation of PB cells.

Using proteomic analysis of G‐CSF mobilised LDN from healthy donors, we identified CD98 as a novel marker for this neutrophil subset, thus obviating the need for density‐gradient separation. Proteomic analysis also revealed enrichment of proteins involved in mitochondrial energy production and enhanced energetic capacity in LDN. This metabolic flexibility is driven by the upregulation of CD98, an essential amino acid transporter. In a nutrient‐depleted environment, CD98 facilitates the accumulation of amino acids that are used by mitochondria to produce energy, which may explain the pathogenicity of LDN at sites of tissue inflammation. Finally, we show that CD98^+^ LDN are present in the PB of SLE patients, and appear to correlate with disease severity, potentially providing a new clinical biomarker in SLE.

## 2 MATERIALS AND METHODS

### 2.1 Study approval

This study was approved by the Human Research and Ethics Committees of Melbourne Health and of the Walter and Eliza Hall Institute of Medical Research (G10/07, G11/07 and G06/02). Written informed consent was obtained from all participants. PB was collected from healthy donors treated with 5 μg/kg G‐CSF twice a day for 4 days at the Victorian Comprehensive Cancer Centre or from healthy non‐G‐CSF‐treated control donors recruited through the Volunteer Blood Donor Registry at the Walter and Eliza Hall Institute of Medical Research. Donors were of any ethnic background, sex or age. PB was collected by venepuncture from SLE patients recruited from Rheumatology Units at the Royal Melbourne Hospital (Victoria, Australia). For this study, eight healthy donors were recruited—four males and four females with an average age of 55.75 years (range 55–72 years). These donors had no known medical conditions. A total of 54 healthy donors treated with G‐CSF (GD) with no other medical conditions were recruited—30 males and 24 females, with an average age of 48.61 years (range 25–66 years). It was not possible to age‐ and sex‐match all healthy donors with the SLE cohort. Patients admitted to hospital or seen in the outpatient clinic for management of SLE were eligible for inclusion and were identified by treating rheumatologists. All patients met 2019 EULAR/ACR classification criteria for SLE. The demographic and clinical characteristics of SLE patients are presented in Table 1.

**TABLE 1:** Systemic lupus erythematosus (SLE) patient cohort

Age	Gender	Disease duration	Clinical features	Autoantibody profile	dsDNA	C′	SLEDAI‐2K	Rx
33	F	15 years	Arthritis, mucocutaneous, neuropsychiatric, renal, serositis cytopenia	ANA (+) Anti‐Ro (+) Anti‐La (+)	↑	N	34	CS, HCQ, RTX
25	M	4 years	Arthritis, mucocutaneous, serositis, kidney, vasculitis	ANA (+) Anti‐b2‐glycoprotein IgG (+) Lupus anticoagulant (+) Anti‐cardiolipin IgG (+)	↑	↓	30	CS, HCQ
32	F	2 months	Arthritis, mucocutaneous, renal, pancytopenia	ANA (+) Anti‐smith (+) Anti‐b2‐glycoprotein IgG (+) Lupus anticoagulant (+) Anti‐cardiolipin IgG (+)	↑	↓	27	CS, AZA, HCQ
25	F	1 month	Arthritis, mucocutaneous	ANA (+) Anti‐Ro (+) Anti‐La (+) Anti‐RNP (+) Anti‐Smith (+)	N	N	6	CS
24	F	13 months	Arthritis, myositis, cytopenia	ANA (+) Anti‐Ro (+) Anti‐La (+) Anti‐RNP (+) Anti‐ribosomal P (+) Rheumatoid factor (+) Anti‐b2‐glycoprotein IgG (+) Anti‐cardiolipin IgG (+)	↑	N	13	CS, HCQ, MMF
46	F	5 years	Vasculitis, mucocutaneous, cytopenia	ANA (+) Anti‐Ro (+) Anti‐La (+) Anti‐b2‐glycoprotein IgG (+) Anti‐cardiolipin IgG (+)	N	N	9	CS, HCQ
63	F	2 months	Arthralgia, renal, serositis, cytopenia	ANA (+)	↑	N	11	CS, RTX
37	F	23 years	Arthralgias, renal, mucocutaneous, cytopenia	ANA (+)	N	↓	19	CS, HCQ, MMF
22	F	3 years	Arthritis, mucocutaneous, autoimmune hepatitis	ANA (+) Anti‐Ro (+) Anti‐La (+) Anti‐RNP (+) Anti‐Smith (+)	↑	↓	14	CS, HCQ, RTX
32	F	4 years	Arthritis, renal, mucocutaneous, serositis, cytopenia	ANA (+)	↑	N	8	CS, HCQ, MMF

Abbreviations: ANA, anti‐nuclear antibody; AZA, azathioprine; C′, complement; CS, corticosteroids; dsDNA, double stranded DNA; F, female; HCQ, hydroxychloroquine; IgG, immunoglobulin G; M, male; MMF, mycophenolate mofetil; RNP, ribonucleotide protein; RTX, rituximab; Rx, medications at the time of venepuncture.

### 2.2 Isolation of neutrophils

Neutrophils from healthy donors, GD or SLE patients were isolated from fresh ethylenediaminetetraacetic acid (EDTA)‐anticoagulated PB using density‐gradient centrifugation on Ficoll, as previously described.^11^ LDN were collected from the peripheral blood mononuclear cell (PBMC) layer, while NDN were collected from the granulocyte–erythrocyte pellet. Brief hypotonic lysis was used to remove red blood cells in both the PBMC and granulocyte–erythrocyte pellet layers before cells were washed in phosphate‐buffered saline (PBS) and centrifuged for 5 min at 200 *g*. Enriched populations of LDN and NDN were obtained using either magnetic bead selection or cell sorting by flow cytometry. For magnetic bead separation, neutrophils were isolated with the EasySep Human Neutrophil Enrichment Kit (StemCell Technologies) according to the manufacturer's instructions. For flow cytometry cell sorting, both the PBMC and granulocyte pellets were resuspended in PBS containing 2% foetal bovine serum (FBS). Cells were incubated with anti‐human CD66b (G10F5), anti‐human CD15 (HI98) and anti‐human CD10 (HI10a) (BD Bioscience) for 30 min at 4°C. CD10^+^ and CD10^−^ NDN and LDN were sorted using a FACS Aria II flow cytometer (Becton Dickinson). Neutrophils were identified based on expression of CD15 and CD66b and the maturation marker CD10 (Figure S1A). Purity of the sorted populations was consistently above 98%, as determined by flow cytometry.

### 2.3 Flow cytometry analysis

For flow cytometry analysis, 3 × 10^5^ cells were incubated in PBS containing 2% FBS and 5 μg/ml human BD Fc block for 15 min. Cells were stained for 30 min in the dark at 4°C with the following antibodies: CD66b (G10F5), CD15 (HI98), CD10 (HI10a), CD16 (3G8), CD11b (ICRF44), CD14 (MφP9), CD62L (DREG‐56) and CD98 (RL388). Data were acquired using the Cytok Aurora spectral flow cytometer and analysed using FlowJo software (Tree Star).

### 2.4 Proteomic analysis

Protein samples were resuspended in 6 M urea, 100 mM dithiothreitol and 100 mM Tris–HCl pH 7.0 and subjected to protein digestion using a filter‐aided sample preparation column.^15^ Peptides were lyophilised to dryness and stored at ‐80°C. Peptides were resuspended in 2% acetonitrile (ACN)/1% formic acid (FA) and separated by reverse‐phase liquid chromatography on an M‐class UHPLC system (Waters, USA) using a 250 mm × 75 mm column (1.6 mm C18, packed emitter tip; Ion Opticks, Australia) with a linear 90‐min gradient at a flow rate of 400 nl/min from 98% solvent A (0.1% FA in Milli‐Q water) to 35% solvent B (0.1% FA, 99.9% ACN). The nano‐UPLC was coupled online to an Impact II mass spectrometer equipped with a CaptiveSpray ionisation source (Bruker Daltonics, Germany) and column oven at 40°C (Sonation, Germany). The Impact II was operated in a data‐dependent mode using a 1.5‐s cycle time, switching automatically between one full‐scan 4 Hz and subsequent MS/MS scans for the remaining time with spectra rate determined using peptide intensity. The instrument was controlled using OtofControl version 4.

Raw files were analysed using MaxQuant (version 1.5.8.3). A database search was performed using the Uniprot Homo sapiens database plus common contaminants, with strict trypsin specificity (allowing up to two missed cleavages). The minimum peptide length was seven amino acids. Carbamidomethylation of cysteine was a fixed modification, while N‐acetylation of protein N‐termini and oxidation of methionine were set as variable modifications. During the MaxQuant main search, precursor ion mass error tolerance was set to 0.006 Da. PSM and protein identifications were filtered using a target‐decoy approach at a false discovery rate (FDR) of 1% with the match between runs and LFQ options enabled. Further analysis was performed using a custom pipeline developed in R (version 3.6.1), which utilises the LFQ intensity values in the MaxQuant output file proteinGroups.txt. Proteins not found in at least 50% of the replicates in one group were removed. Missing values were imputed using a random normal distribution of values with the mean set at mean of the real distribution of values minus 1.8 standard deviation (SD), and SD of 0.3 times the SD of the distribution of the measured intensities. The probability of differential site modification expression between groups was calculated using the limma R package (version 3.4.2). Probability values were corrected for multiple testing using the Benjamini–Hochberg method.

### 2.5 Bioinformatic analysis of proteomic data

Prior to analysis, all rows in the data sheet labelled either ‘Reverse’ or ‘Potential contaminant’ were removed. The data were then formatted such that rows were proteins and columns were samples, and all proteins whose intensity was 0 were replaced with not available intensity. Lowly abundant proteins were filtered from the data. All retained proteins were required to have an intensity greater than or equal to 1 in at least three samples, leaving a total of 3847 unique proteins. Following filtering, quantile normalisation was applied to the intensity data. Differential abundance analysis between the LDN and NDN neutrophils was undertaken using the limma software package (version 3.42.2). The correlation between samples from the same donor was first estimated using limma's duplicate correlation function. Differentially abundant proteins were then identified using linear models that incorporated the aforementioned correlation estimate and blocked on sample donor, together with robust empirical Bayes moderated t‐statistics with a trended prior variance (robust limma‐trend pipeline). The variance of those proteins that were missing in all samples from one group was adjusted by re‐fitting the data for those proteins only, and replacing the variance estimates in the initial model fit. This was done for each group. The Benjamin–Hochberg procedure was used to keep the FDR below 5%. Subsequent pathway analyses were performed using limma's goanna and kegga functions. Volcano and multi‐dimensional scaling (MDS) plots were created using limma's volcano and plotMDS functions, respectively. Heatmaps were generated using the pheatmap software package.

### 2.6 Metabolomic analysis

For the metabolomics study, 1 × 10^7^ NDN and LDN were isolated using flow cytometry. Samples were extracted for liquid chromatography–mass spectrometry (LC–MS) analysis by adding 180 μl of ice‐cold 2:2:1 ACN:methanol:Milli‐Q water containing 2 μM ^13^C‐sorbitol, ^13^carbon (C),^15^nitrogen (N)‐valine, ^13^C,^15^N‐Adenosine monophosphate (AMP) and ^13^C,^15^N‐Uridine monophosphate (UMP) as internal standards. Cell supernatants were collected and centrifuged at 14 000 *g* for 10 min (at 4°C) to remove cell debris. Metabolite analysis was performed on an Agilent quadrupole time‐of‐flight mass spectrometer (Q‐TOF MS) (Agilent Technologies) with chromatographic separation on an Agilent 1200 series HPLC system (Agilent Technologies). Metabolites were separated by injecting the sample, onto a Merck SeQuant ZIC‐HILIC column (150 mm × 4.6 mm, 5 μm particle size) maintained at 25°C, using a binary gradient consisting of solvent A: 20 mM ammonium carbonate (pH 9.0; Sigma–Aldrich) and solvent B: 100% ACN. The gradient run was as follows: time (*t*) = 0.0 min, 80% B; *t* = 0.5 min, 80% B; *t* = 15.5 min, 50% B; *t* = 17.5 min, 30% B; *t* = 18.5 min, 5%; *t* = 21.0 min, 5% B; *t* = 23–33 min, 80% at a solvent flow rate of 300 μl/min. MS detection was performed on an Agilent 6545 Q‐TOF MS operating in negative electrospray ionisation mode as described previously.^16^ Data were acquired in centroid mode with a scan range of 60–1200 m/z and an acquisition rate of 1.5 spectra/s. Samples were analysed in a single analytical batch and randomised with a pooled biological quality control (pbQC) every five samples to monitor instrument performance. Data were analysed using Agilent Mass Hunter Quantitative Analysis Software B. Level 1 metabolite identification, according to the Metabolite Standard Initiative,^17^ was based on matching accurate mass, retention time and MS/MS spectra to the 550 authentic standards in the Metabolomics Australia (MA) in‐house library.

### 2.7 Bioinformatic analysis of metabolomic data

Prior to analysis, pbQCs were removed, and annotation from the Human Metabolome Database was added using the hmdbQuery software package. Quantile normalisation was then applied to the data. Differential analyses between the cell types were then undertaken using the limma software package (version 3.44.3). The correlation between samples from the same donor was first estimated using the limma duplicate correlation function. Linear models were then fitted to each metabolite, incorporating the correlation estimate (blocking on donor), as well as an adjustment for sample batch. Robust empirical Bayes t‐statistics with a trended prior variance (robust limma‐trend pipeline) were then applied to identify significant metabolites. The Benjamini–Hochberg method was used to keep the FDR below 5%. Successive pathway analysis of the small molecule pathway database was performed using limma's kegga function. A heatmap was generated using the pheatmap software package.

### 2.8 Metabolic analysis of neutrophil subpopulations

A total of 4 × 10^6^ neutrophils were plated in quadruplicate into a Seahorse XF96 Cell Culture Microplate (Agilent Technologies) coated with Cell‐Tak (Agilent Technologies) and centrifuged for 1 min at 100 *g*, followed by deceleration without braking. Neutrophils were plated in Seahorse XF Roswell Park Memorial Institute (RPMI) medium containing 1 mM glutamine and 10 mM glucose and incubated in a CO_2_‐free environment at 37°C for 1 h. In some cases, glucose‐ and glutamine‐free medium was used to examine the effects of nutrient deprivation on cellular metabolism. The oxygen consumption rate and extracellular acidification rate (ECAR) were measured using the adenosine triphosphate (ATP) Rate kit, Mito Stress Test kit and Glyco Stress Test kits on the XF96 Extracellular Flux Analyser (Seahorse Bioscience) according to the manufacturer's instructions. To determine the effect of CD98 inhibition on mitochondrial metabolism, neutrophils were incubated for 2 h in the presence of 1 μM JPH203 (Tocris) or vehicle control Dimethylsulfoxide (DMSO) before a Mito Stress Test assay was performed.

### 2.9 Assessment of mitochondrial mass and membrane potential

LDN and NDN were incubated with anti‐human CD66b and anti‐human CD10 for 30 min in the dark at 4°C and washed twice in PBS containing 2% FBS. Assessment of mitochondrial mass and membrane potential was performed by staining neutrophils with either MitoTracker Green (5 nM) or tetramethylrhodamine, ethyl ester (TMRE; 40 nM) in complete Seahorse XF RPMI medium (1 mM glutamine and 10 mM glucose) for 15 min at room temperature. Data were acquired using the Cytok Aurora spectral flow cytometer and analysed using FlowJo software (Tree Star).

### 2.10 Quantification of cell death

Cells were seeded into 48‐well plates in phenol‐free RPMI 1640 medium supplemented with 10% foetal calf serum (FCS) at a density of 6 × 10^4^ cells per well. Cells were allowed to equilibrate under humidified 10% CO_2_ at 37°C for 1 h before the medium was supplemented with 1:500 dilution of AlexaFluor488‐conjugated Annexin V (ThermoFisher Scientific) and 0.5 μg/ml propidium iodide (PI) (Sigma). Cells were treated with combinations of the following agonists/antagonists: 100 ng/ml recombinant human TNF (produced in‐house^18^), 500 nM Smac‐mimetic/compound A (Tetralogic Pharmaceuticals^19^), 5 μM IDN‐6556 (Idun Pharmaceuticals), 100 μg/ml cycloheximide (Sigma), 1 μM ABT‐737 (Abbott) and 0.1 μM Mcl‐1 inhibitor (also known as S63485; SYNthesis MedChem). Cells were transferred to an IncuCyte S3 System (Essen Bioscience) and imaged over time using the 10× objective and the default bright‐field, green and red channel settings. The number of Annexin V/PI‐positive cells per mm^2^ over time was quantified using IncuCyte S3 v2018A software (Essen Bioscience).

### 2.11 Quantification of plasma IFNα

EDTA‐anticoagulated blood was collected, and plasma was isolated by centrifugation at 1500 *g* for 15 min, followed by centrifugation at 3500 *g* in a fresh tube for 15 min to remove platelets. Plasma samples were stored at −80°C until analysis. IFNα levels were measured using the Human IFN‐α Flex Set (BD Bioscience) according to the manufacturer's instructions.

### 2.12 Luminex to measure lipopolysaccharides‐induced cytokine production

Pure populations of LDN and NDN were isolated, and cells were resuspended at a concentration of 2 × 10^6^ cells/ml in RPMI 1640 medium supplemented with 10% FCS. For each condition, 1 × 10^6^ of each neutrophil subset was cultured in the presence or absence of 10 ng/ml lipopolysaccharides (LPS, Sigma). Cell culture medium was collected after 16 h and the concentrations of cytokines and chemokines were determined using a Luminex assay (R&D Systems), which has a lower limit of detection of 3.2 pg/ml.

### 2.13 Quantification of interleukin‐6 production using ELISpot

LPS‐induced interleukin‐6 (IL‐6) secretion was assessed using an ELISpot assay (Mabtech, Nacka Strand, Sweden). For each neutrophil subset, 1 × 10^4^ cells per well were plated in triplicate in RPMI 1640 medium supplemented with 10% FCS and 10 ng/ml LPS (Sigma). Wells were visualised using a computerised ELISpot reader system (AID iSPOT reader system), and the production of IL‐6 was calculated using iSPOT software.

### 2.14 Statistics

Differences between LDN and NDN were assessed using a one‐way analysis of variance (ANOVA). For repeated measures, the Tukey's multiple comparisons post hoc test or two‐way ANOVA with Dunnett's multiple comparisons test was used to compare differences between the groups. For other experiments, differences between groups were determined using unpaired Student's *t*‐tests. For data obtained from SLE patients, non‐parametric statistical tests, including Mann–Whitney and Spearman's correlation, were applied. All analyses were performed using GraphPad Prism version 9 (GraphPad Software). Data are shown as means ± standard error of mean unless stated otherwise. Levels of statistical significance are expressed as *p*‐values: ^*^ *p* < .05, ^**^ *p* < .01 and ^***^ *p* < .001.

### 2.15 Data and materials availability

The MS proteomics data have been deposited to the ProteomeXchange Consortium via the PRIDE partner repository, with the dataset identifier PXD023952.

## 3 RESULTS

### 3.1 G‐CSF mobilised LDN into PB

**FIGURE 1:**
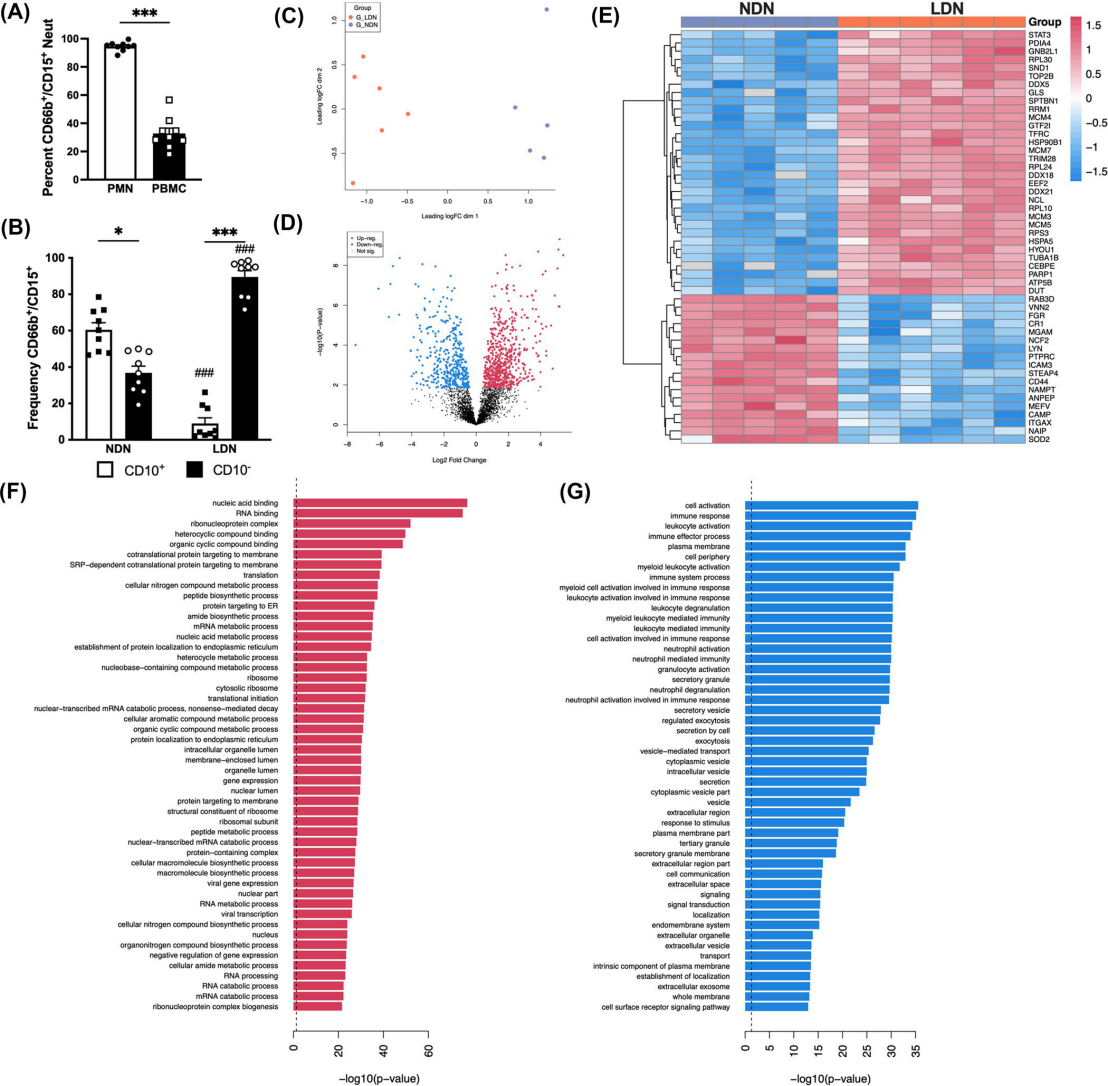
Low‐density neutrophils (LDN) have a different proteomics profile compared to normal‐density neutrophils (NDN). (A) The proportion of CD66b^+^/CD15^+^ neutrophils within the polymorphonuclear neutrophil (PMN) (black) and peripheral blood mononuclear cell (PBMC) (white) layers after density separation of peripheral blood (PB) from GD (*n* = 9). (B) Frequency of CD10^+^ (white) and CD10^−^ (black) neutrophils within the PMN and PBMC layers from healthy donors treated with granulocyte colony‐stimulating factor (G‐CSF) (GD) (*n* = 9). (C) Principal component analysis (PCA) of the proteomic profiles of NDN versus LDN (*n* = 6). (D) Quantitative analysis of NDN and LDN, with the volcano plot representing relative protein abundance changes (*n* = 6). (E) Heatmap representing the top 50 differentially expressed proteins across both groups, where each column represents an individual donor. Gene Ontology (GO) analysis identified biological processes significantly enriched in either LDN (F) or NDN (G) (*n* = 6). Data are mean ± standard error of mean (SEM). ^*^ *p* < .05, ^***^ *p* < .001, one‐way analysis of variance (ANOVA)

Consistent with previous reports, LDN co‐purified with mononuclear cells during density‐gradient centrifugation of PB from GD (Figures 1A and S1A for representative dot blots).^11^ Morphological analysis revealed that NDN contained both mature and immature neutrophils, while LDN were predominantly immature‐looking cells, with a small proportion of mature neutrophils (Figure S1A). Consistent with these findings, most LDN lacked the neutrophil maturation marker CD10, whereas NDN contained both CD10^+^ mature and CD10^−^ immature cells (Figure 1B).^11^ The expression of neutrophil maturation and activation markers was assessed on both mature and immature neutrophils within each density layer (Figure S1C displays representative histogram showing marker expression levels). Mature and immature NDN expressed similar levels of all neutrophil markers examined (Figure S1D). While LDN expressed more CD66b than NDN regardless of maturation status, immature LDN had a significant increase in the median fluorescence intensity (MFI) of CD66b compared to mature LDN. Mature LDN had similar levels of CD15, CD11b and CD14 compared to NDN, and there was a significant reduction in CD16 and CD62L. As CD16 and CD62L decrease upon neutrophil activation,^20^ mature neutrophils in the PBMC layer likely represent an activated pool of cells, consistent with previous reports.^21^ Strikingly, the expression of almost all markers examined was significantly different in immature LDN compared to the other neutrophil subsets. While immature LDN expressed significantly increased CD66b, expression of CD16, CD11b, CD14 and CD62L was significantly reduced compared to both mature neutrophils found in the PBMC fraction, as well as immature and mature NDN. In a preliminary experiment, expression of CD45 was downregulated on LDN compared to NDN, matching the proteomics analysis (Figure S1C). This was unexpected given previous literature,^22^ but would be in keeping with less mature morphology.

### 3.2 LDN have a significantly different proteome compared to NDN

Previous studies have focussed on transcriptional differences between LDN and NDN, with only one examining changes to the proteome.^23^ Given that neutrophils are relatively transcriptionally quiescent, we conducted a whole‐cell proteomics analysis of LDN and NDN to better understand functional differences between these two subsets. MS analysis of LDN or NDN from GD detected 3904 proteins across both subsets (Table S1) and was generally in keeping with other studies examining the neutrophil proteome.^23^ An MDS plot revealed two distinct clusters corresponding to each neutrophil subset (Figure 1C). A total of 973 proteins were differentially expressed between the two subpopulations, with 581 proteins significantly enriched in LDN and 392 downregulated in LDN, compared to NDN (Figure 1D). The top 50 most differentially expressed proteins are shown in a heatmap (Figure 1E). STAT3 was upregulated in LDN compared to NDN, most likely reflecting higher expression of the G‐CSF receptor in immature neutrophils, leading to enhanced G‐CSF signalling.^24^ We also found increased expression of DDX family members 5, 18 and 21. DDX proteins are required for RNA metabolism and facilitate gene expression.^25^ Upregulation of DDX proteins in LDN is in keeping with a recent report demonstrating that LDN are more transcriptionally active than NDN.^26^ Gene Ontology (GO) analysis revealed that proteins enriched in LDN were predicted to be involved in protein production and transport, cell cycle regulation and metabolic processes (Figure 1F and Table S2). Proteins enriched in NDN are predicted to be involved in immune cell activation, neutrophil degranulation and phagocytosis (Figure 1G and Table S3).

### 3.3 Changes in intracellular metabolites in LDN

**FIGURE 2:**
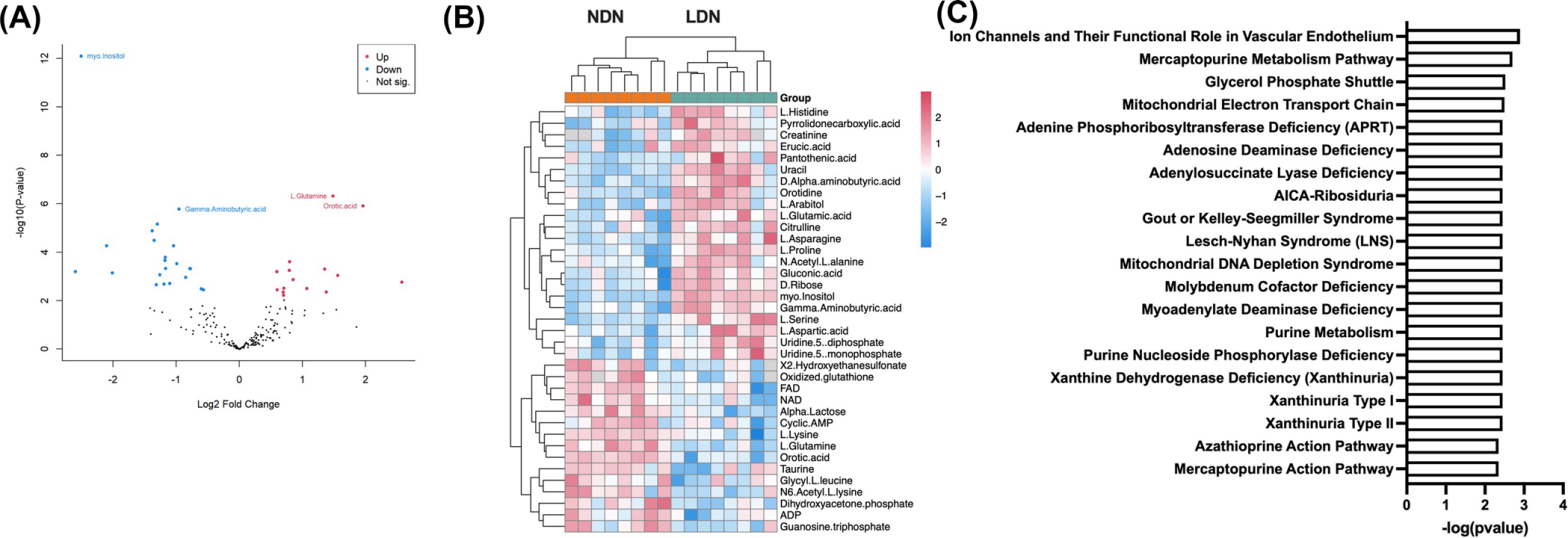
Metabolite changes in CD98^+^ low‐density neutrophils (LDN) compared to CD98^−^ normal‐density neutrophils (NDN). (A) Quantitative analysis of NDN and LDN, where the volcano plot represents relative protein abundance changes. Red represents metabolites upregulated in NDN and blue represents metabolites upregulated in LDN (*n* = 6). (B) Heatmap of differentially expressed metabolites between LDN (green) and NDN (blue), where each column represents an individual donor (*n* = 6). (C) Pathway enrichment analysis of significantly different metabolites in LDN compared to NDN (*n* = 6). Peripheral blood (PB) from six individual granulocyte colony‐stimulating factor (G‐CSF) donors was analysed

Our proteomics analysis found over 770 proteins differentially expressed in LDN compared to NDN that were associated with cellular metabolic processes (Table S2). We therefore conducted a metabolomic analysis. A total of 205 metabolites occurred across both subsets, but 37 metabolites were differentially expressed in LDN compared to NDN (Figure 2A,B). These metabolites were associated with ion channels, mercaptopurine metabolism and the glycerol phosphate shuttle (Figure 2C). Metabolites associated with the mitochondrial electron transport chain were also enriched in LDN, including adenosine diphosphate, dihydroxyacetone phosphate, flavin adenine dinucleotide and nicotinamide adenine dinucleotide.

### 3.4 CD98 is expressed by LDN in PB of GD

**FIGURE 3:**
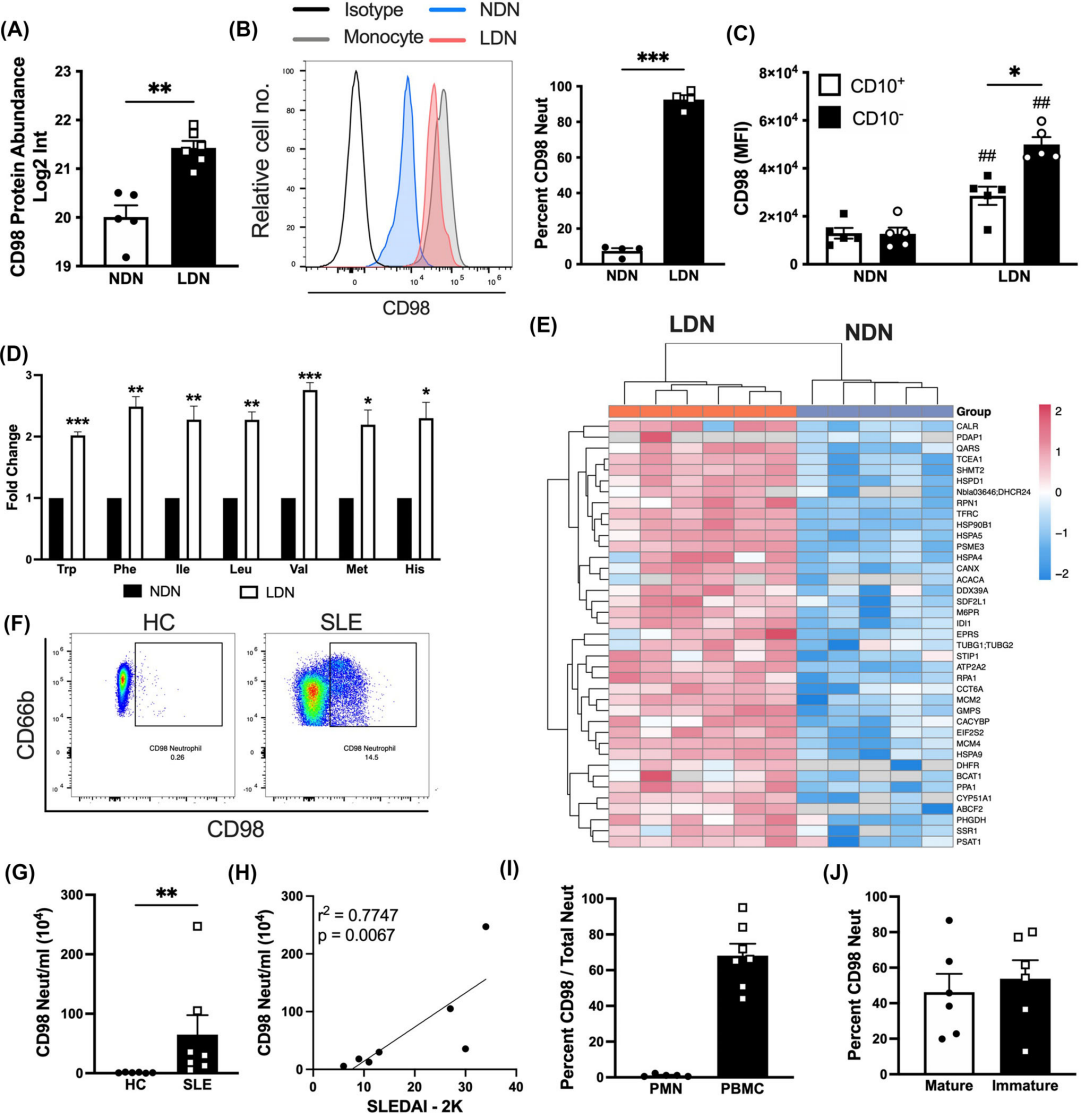
CD98 defines a subset of low‐density neutrophils (LDN) in the peripheral blood (PB) of healthy donors treated with granulocyte colony‐stimulating factor (G‐CSF) (GD) and systemic lupus erythematosus (SLE) patients. (A) Abundance of CD98 in whole‐cell lysates measured using proteomics and mass spectrometry (*n* = 6). (B) Membrane expression of CD98 on normal‐density neutrophils (NDN) (blue), LDN (red) and monocytes (grey) displayed as a representative histogram, with percentage of CD98^+^ cells (*n* = 5). LDN stained with the appropriate isotype control antibody is shown in white. (C) Levels of CD98 on CD10^+^ (black) and CD10^−^ (white) NDN and LDN, represented as MFI (*n* = 5). (D) Fold change in intracellular levels of essential amino acid content relative to NDN (*n* = 6). (E) A heatmap of proteins linked to the mammalian target of rapamycin complex 1 (MTORC1) signalling pathway enriched in LDN (*n* = 6). (F) Representative dot blots displaying CD98 expression in neutrophils from healthy controls (*n* = 5) and SLE patients (*n* = 7). (G) Quantification of CD98^+^ neutrophils per ml peripheral blood (PB) in healthy controls (*n* = 5) and SLE patients (*n* = 7). (H) Correlation between CD98^+^ neutrophils in PB and Systemic Lupus Erythematosus Disease Activity Index 2000 (SLEDAI‐2K) in SLE patients (*n* = 7). (I) Frequency of CD98^+^ neutrophils within the polymorphonuclear neutrophil (PMN) (white) and peripheral blood mononuclear cell (PBMC) (black) layers following density‐gradient separation of PB from SLE patients (*n* = 7). (J) Maturation status of CD98^+^ neutrophils, determined by the expression of CD10 in PB of SLE patients (*n* = 7). Data are mean ± standard error of mean (SEM). ^#/*^ *p* < .05, ^##/**^ *p* < .01, ^###/***^ *p* < .001, Student's *t*‐test or one‐way analysis of variance (ANOVA). Correlation between CD98^+^ neutrophils and SLEDAI was assessed using Spearman's correlation

One aim of this study was to search for a phenotypic marker of LDN without the need for gradient density separation of PB cells. GO analysis identified 300 differentially expressed plasma membrane proteins in LDN compared to NDN (Tables S2 and S3). We focussed on membrane proteins upregulated by at least twofold in LDN compared to NDN. In order to facilitate identification by flow cytometry, we selected proteins with low/absent expression on NDN. Using these criteria, CD98hc (SLC3A2, Figure 3A) was identified as the top candidate phenotypic marker for LDN. CD98 is a type II transmembrane protein that dimerises with several L‐type amino acid transporters to facilitate uptake of essential amino acids.^27^ Monocytes and activated lymphocytes express high levels of CD98, but neutrophils typically have minimal expression.^28^ In line with previous studies, NDN expressed minimal CD98 (Figure 3B). In contrast, LDN had significantly increased expression of membrane CD98, with levels comparable to those of monocytes (Figures 3B and S2A). An average of 92.5% of LDN from GD expressed CD98, compared to only 7.4% NDN (Figure 3B). As LDN in disease can include both mature and immature neutrophils,^3^ we confirmed that CD98 was upregulated in both mature and immature LDN. Of note, immature LDN expressed more cell surface CD98 than LDN. Little expression of CD98 was observed on NDN regardless of maturation status (Figure 3C).

CD98 plays a role in the transport of essential amino acids, so MS analysis was used to quantify the amino acid content of each neutrophil subset (Figures 3D and S2B). LDN contained more essential amino acids, including large hydrophobic (methionine and histidine), branched chain (valine, leucine and isoleucine) and aromatic (phenylalanine and tryptophan) amino acids compared with NDN (Figure 3D). The levels of other intracellular amino acids were not significantly different between the two neutrophil subsets (Figure S2B). CD98‐mediated amino acid transport is also known to induce activation of the mammalian target of rapamycin (mTOR) signalling pathway,^28^ and we observed a significant enrichment of proteins involved in this pathway in LDN compared to NDN (Figure 3E).

### 3.5 LDN are expanded in PB of SLE patients

We sought to determine if the CD98^+^ LDN population could also be identified in patients with SLE because previous publications have demonstrated that LDN are significantly expanded in the PB of these patients^5^, ^10^, ^29^, ^30^ (Figure S2C). PB was collected from patients with active SLE or non‐G‐CSF‐treated healthy individuals as controls (Table 1). Very few CD98^+^ neutrophils were detected in the blood of healthy individuals, while SLE patients had an increased number and percentage of this subset (Figures 3F,G and S2C,D). Strikingly, the number of CD98^+^ neutrophils in PB of SLE patients correlated with disease activity scores as measured by SLEDAI‐2K in a small pilot study (Figure 3H). Previous reports suggest that LDN in SLE are associated with the type I IFN signature and have an enhanced capacity to synthesise IFNα.^5^, ^30^ Despite the correlation with SLEDAI‐2K, we observed no correlation between IFNα and the burden of CD98^+^ neutrophils in PB, albeit in this small cohort of patients (Figure S2E).

Consistent with GD, CD98^+^ neutrophils were low density and almost exclusively located within the PBMC layer following density‐gradient separation of PB (Figure 3I). However, not all LDN expressed CD98 in SLE, with (on average) 68.12% of CD98‐positive LDN (Figure 2I). This suggests that CD98^+^ neutrophils could represent a distinct subset of LDN. We next conducted an analysis of the size and granularity of CD98^+^ and CD98^−^ neutrophils from SLE patients using flow cytometry. CD98^+^ neutrophils were comparable in size but more granular than CD98^−^ neutrophils, as indicated by an increase in mean side scatter (Figure S2F,G). In keeping with the results obtained from GD, CD98^+^ neutrophils comprised both mature and immature neutrophils, as determined by CD10 expression (Figures 2J and S2G). CD98^+^ neutrophils from SLE patients had heterogenous expression of CD66b. To better understand this heterogeneity, we analysed CD98^+^ neutrophils based on high, intermediate, and low CD66b expression (Figure S2I). We observed no difference in the size or granularity of CD98^+^ neutrophils regardless of CD66b expression, and each population contained mostly immature CD10^−^ neutrophils.

### 3.6 LDN have enhanced mitochondrial bioenergy capacity

**FIGURE 4:**
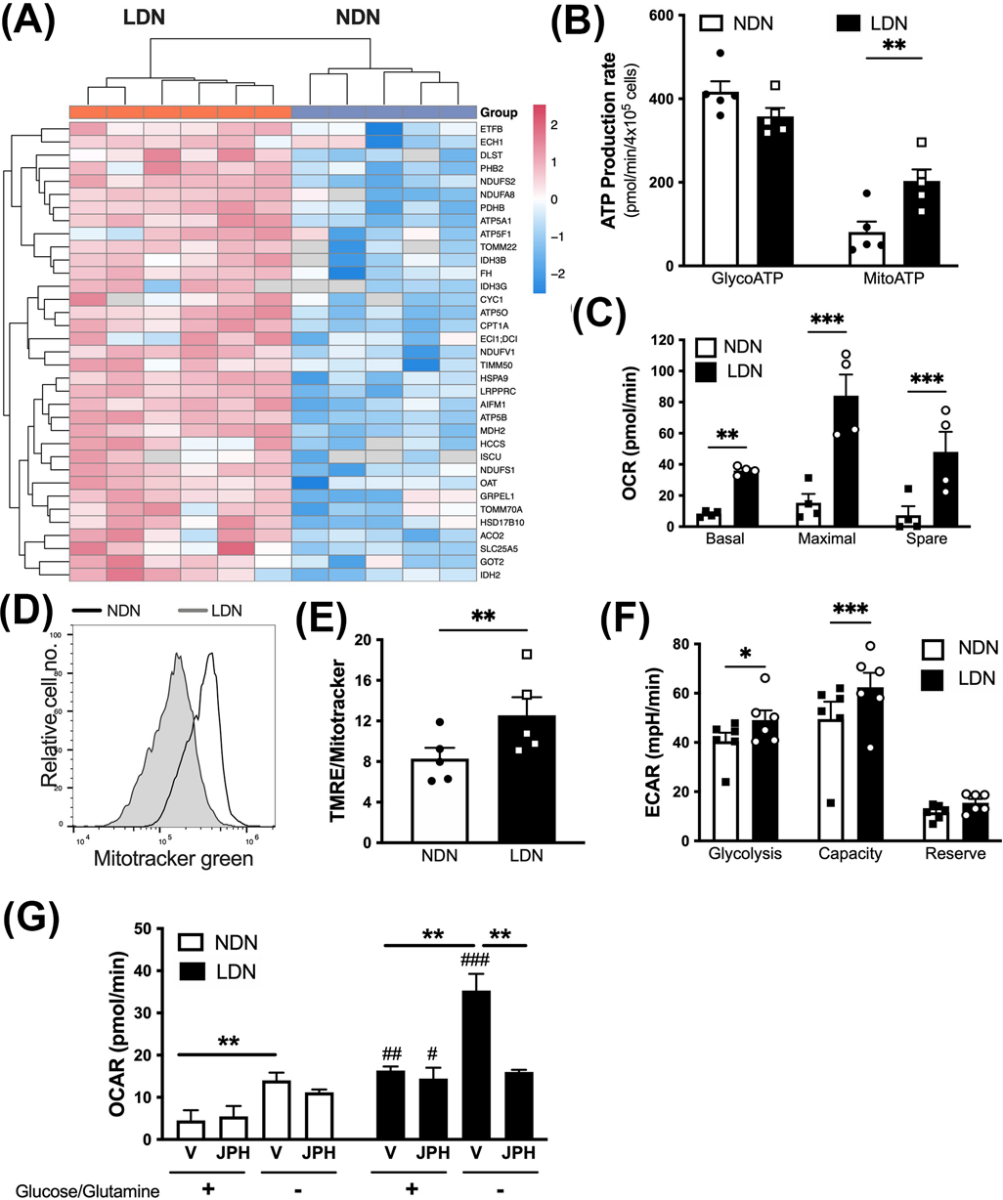
CD98^+^ low‐density neutrophils (LDN) have increased mitochondrial respiratory capacity compared to CD98^−^ normal‐density neutrophils (NDN). (A) Heatmap of proteins linked to oxidative phosphorylation (OXPHOS) enriched in LDN. (B) Rate of adenosine triphosphate (ATP) produced through glycolysis (GlycoATP measured by extracellular acidification rate, ECAR) or mitochondria (MitoATP measured by oxygen consumption rate, OCR) in each neutrophil subset (*n* = 4). (C) A mitochondrial stress test was performed to measure basal, maximal and spare mitochondrial respiratory capacity (*n* = 4). (D) Representative histogram of MitoTracker Green staining of the neutrophil subsets. (E) Relative tetramethylrhodamine, ethyl ester (TMRE)/MitoTracker Green ratio (*n* = 5). (F) Glycolysis stress measuring glycolysis, glycolytic capacity and glycolytic reserve (*n* = 6). (G) NDN and LDN were treated with JPH203 or vehicle in media, with or without glucose and glutamine. A mitochondrial stress test was performed to measure basal mitochondrial respiration (*n* = 3). Data are mean ± standard error of mean (SEM). ^#/*^ *p* < .05, ^##/**^ *p* < .01, ^###/***^ *p* < .001, Student's *t*‐test or one‐way analysis of variance (ANOVA)

The proteomic data revealed enrichment for oxidative phosphorylation (OXPHOS) proteins in LDN relative to NDN (Figure 4A and Table S2). We used LDN and NDN isolated from GD to explore the functional differences between these two neutrophil subsets. Bioenergetic analysis revealed that the levels of ATP generated through glycolysis were equivalent between neutrophil subsets. However, LDN produce more ATP compared with NDN (Figure S3A) and this was driven by an increase in ATP produced through the mitochondria (Figure 4B). Further evaluation of mitochondrial respiration showed an increase in basal OXPHOS in LDN, and both the maximal respiratory rate and spare respiratory capacity were also increased (Figures 4C and S3B,C). Surprisingly, LDN had a significantly decreased mitochondrial mass compared to both NDN, although no difference in the mitochondrial membrane potential was detected (Figures 4D and S3D,E). We therefore assessed the relative ratio of TMRE to MitoTracker Green staining and found that LDN had a significantly higher ratio compared to NDN (Figure 4E). This finding suggests that while LDN have less mitochondria than NDN, LDN mitochondria are more active.

While no difference in the ECAR was observed under normal conditions, a glucose stress test was conducted to examine glycolysis in more depth. Glucose‐starved LDN had a greater increase in glycolysis relative to NDN following the reintroduction of glucose into the medium (Figures 4F and S3F–H). The glycolysis stress test also revealed that the rate of glycolysis and reserve glycolytic capacity were significantly increased in LDN compared to NDN (Figure 4F). Mitochondrial respiration in LDN was significantly higher compared to NDN in nutrient‐low conditions, and this effect was maintained even after the addition of glucose (Figure S3I). As expected, the relative levels of mitochondrial respiration decreased in response to the addition of glucose in both neutrophil subsets; however, the relative decrease was smaller in LDN compared to NDN (Figure S3J,K). Taken together, our results suggest that LDN are more metabolically flexible and better equipped to adapt to stressed or nutrient‐deficient environments.

### 3.7 CD98 drives the metabolic flexibility of LDN

Previous reports in murine LDN suggest that metabolic flexibility in a nutrient‐depleted environment is due to the ability to use amino acids as an alternative fuel source for metabolically demanding functions.^10^ Amino acids are also known to activate the mTOR, a signalling pathway that facilitates cellular growth, activation, autophagy and metabolism.^31^ To determine if CD98‐mediated transport of amino acids was responsible for this enhanced energetic capacity of human LDN, we used the small molecule inhibitor JPH203.^32^ JPH203 is a selective CD98 inhibitor that blocks amino acid uptake and has been used both in vitro and in vivo, including in phase 1 studies in cancer.^32^, ^33^ In the presence of glucose and glutamine, basal mitochondrial respiration and maximal respiratory capacity were increased in LDN compared to NDN. Under these conditions JPH203 had no effect, most likely because both subsets preferentially rely on glycolysis to produce ATP (Figure 4G). Lack of glucose/glutamine increased the basal and maximal respiratory rates in both neutrophil subsets. Under these conditions, LDN generated significantly more energy through mitochondrial respiration. Strikingly, treatment with JPH203 during nutrient deprivation completely inhibited increased mitochondrial energy production in LDN (Figure 4G). In contrast, JPH203 had no effect on the ability of NDN to generate energy through OXPHOS. Taken together, our results reveal that upregulation of CD98 on LDN is responsible for metabolic flexibility when LDN encounter a nutrient‐depleted environment.

### 3.8 LDN are resistant to spontaneous apoptosis, and this is driven by inhibitors of apoptosis proteins

**FIGURE 5:**
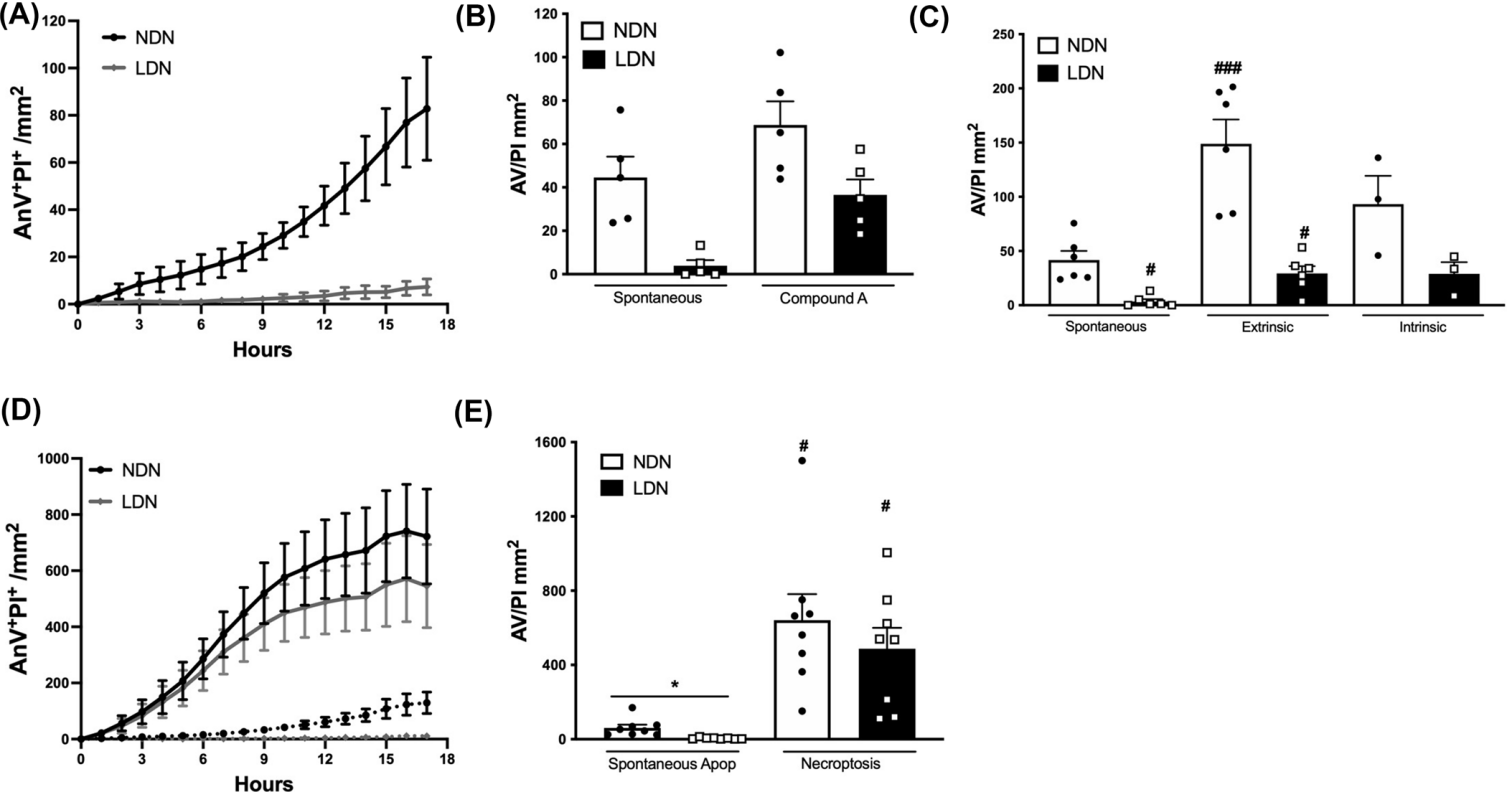
CD98^+^ low‐density neutrophils (LDN) are relatively resistant to apoptosis, but equally susceptible to necroptosis. Cell death was evaluated using the IncuCyte S3 system. (A) Spontaneous apoptosis of normal‐density neutrophils (NDN) (white) and LDN (black) was measured over 18 h (*n* = 5). (B) Quantification of apoptosis after 12 h induced by Smac‐mimetic compound A (*n* = 5). (C) Spontaneous apoptosis (*n* = 5), extrinsic apoptosis (stimulated with tumor necrosis factor [TNF] and a Smac‐mimetic, *n* = 6) or intrinsic apoptosis (stimulated with ABT‐737 and an Mcl‐1 inhibitor, *n* = 3) quantified after 12 h. (D) Both neutrophil subsets were treated with a necroptotic stimulus (TNF, Smac‐mimetic and IDN‐6556 [TSI]) and cell death was measured over 18 h using the IncuCyte S3 System (*n* = 8). (E) The level of necroptotic cell death at 12 h was quantified (*n* = 8). Data are mean ± standard error of mean (SEM). ^*^ *p* < .05, ^**^ *p* < .01, ^***^ *p* < .001, one‐way analysis of variance (ANOVA)

Our proteomics analysis found 158 proteins associated with cell death were differentially expressed in LDN compared to NDN. Neutrophils undergo apoptosis, even in the absence of stimuli, and this helps regulate neutrophil homeostasis.^34^ To further characterise apoptotic capacity, we assessed spontaneous apoptosis or apoptosis in response to stimuli. As expected, NDN rapidly underwent apoptosis. Strikingly, LDN were highly resistant to apoptotic cell death and had little annexin V or PI staining after 18 h (Figure 5A,B). Treatment of LDN with a Smac‐mimetic that targets inhibitors of apoptosis proteins (IAP)^35^ restored apoptosis to levels comparable to those of NDN, indicating that these proteins are likely responsible for resistance to spontaneous cell death. We also investigated whether LDN were resistant to death by extrinsic or intrinsic apoptosis. Treatment with TNF and Smac‐mimetic to induce extrinsic apoptosis significantly increased the level of death in both neutrophil subsets, but LDN underwent less extrinsic apoptosis compared to NDN (Figures 5C and S4A,B). ABT‐737 and Mcl‐1 inhibitors also induced less intrinsic apoptosis, indicating that LDN are more resistant to all forms of apoptosis, which may translate to a longer life span in vivo than NDN.

### 3.9 LDN and NDN readily undergo necroptosis

Apoptosis is important in controlling neutrophil‐mediated inflammation as it is non‐lytic and elicits an anti‐inflammatory response that promotes the resolution of inflammation.^36^ In contrast, necroptosis is a lytic, proinflammatory form of cell death induced by death receptors, IFNs, Toll‐like receptors, and intracellular RNA and DNA sensors. This form of cell death is caspase independent and requires the proteins receptor‐interacting serine/threonine‐protein kinase 3 (RIPK3) and mixed lineage kinase domain‐like (MLKL).^36^ To investigate necroptotic capacity, both neutrophil subsets were treated with the widely used necroptosis‐inducing stimulus TNF, Smac‐mimetic and IDN‐6556 (TSI^37^). In response to TSI, LDN and NDN underwent equivalent degrees of necroptosis. While LDN appeared to have a small reduction in the rate of appearance of AnV^+^/PI^+^ double‐positive (Figure 5D,E) or PI^+^ single‐positive neutrophils (Figure S4D), this was not statistically significant.

### 3.10 LDN produce increased proinflammatory cytokines and chemokines in response to LPS

**FIGURE 6:**
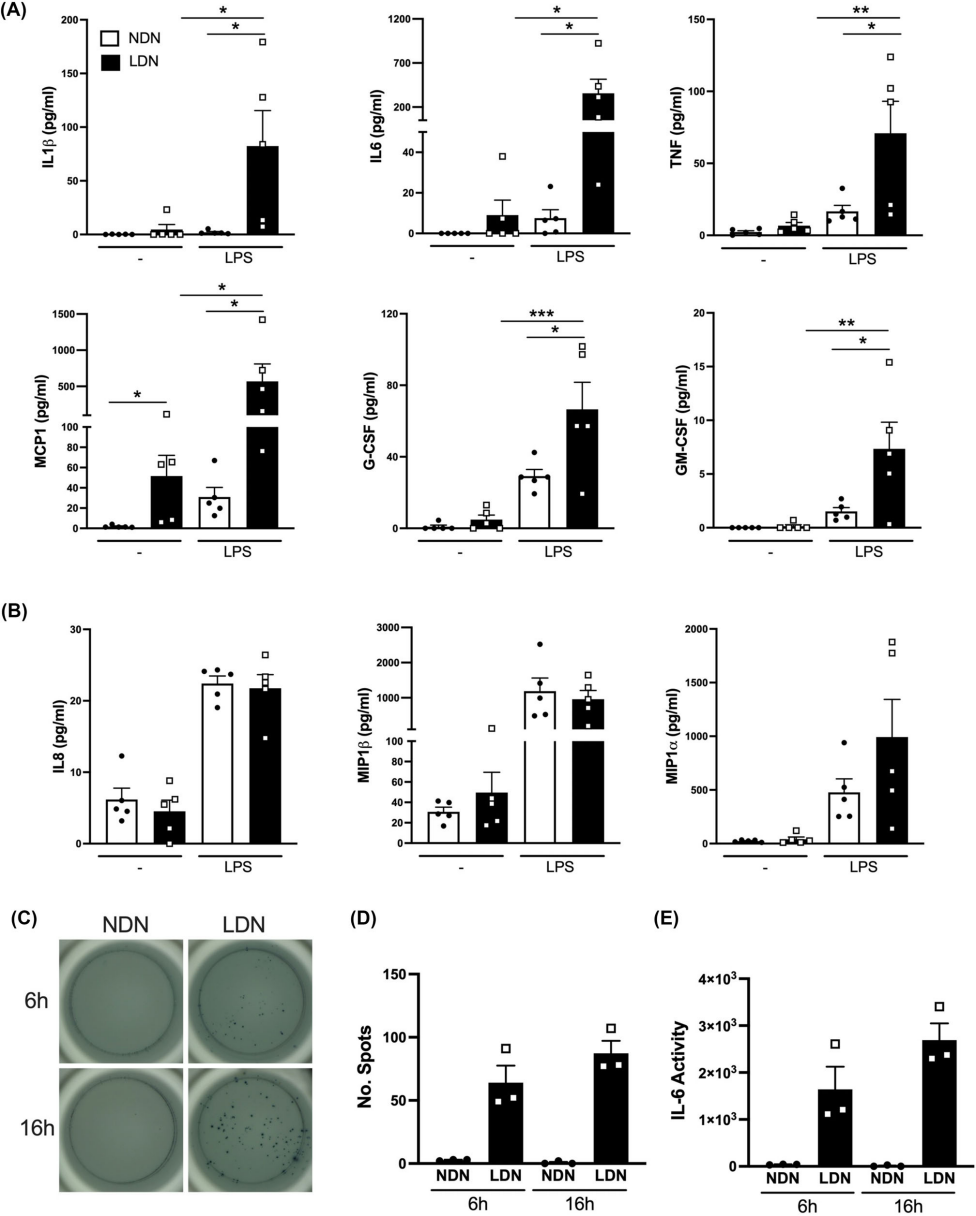
CD98^+^ low‐density neutrophils (LDN) generate more proinflammatory cytokines and chemokines in response to lipopolysaccharides (LPS). (A and B) Normal‐density neutrophils (NDN) (white) and LDN (black) were stimulated for 16 h with LPS (10 ng/ml) and cytokine secretion was measured in the media using a Luminex array (*n* = 5). (C) Representative images of LPS‐induced interleukin‐6 (IL‐6) secretion using ELISpot assays. Quantification of (D) the number of cells secreting IL‐6 and (E) secretory activity. Data represent three replicates from one healthy donor treated with granulocyte colony‐stimulating factor (G‐CSF) (GD). Data are mean ± standard error of mean (SEM). ^*^ *p* < .05, ^**^ *p* < .01, ^***^ *p* < .001, one‐way analysis of variance (ANOVA)

GO analysis revealed that proteins associated with the cellular response to LPS and the Toll‐like receptor 4 signalling pathway are also differentially regulated in LDN compared to NDN. To examine this further, the secretion of cytokines and chemokines was measured in both neutrophil subsets at basal levels and after stimulation with LPS. Under basal conditions, LDN produced significantly more monocyte chemoattractant protein‐1 (MCP‐1) compared to NDN (Figure 5A), and there was a trend towards increased production of IL‐1β, IL‐6, TNF and G‐CSF, although this trend did not reach statistical significance. Treatment of both neutrophil subsets with LPS induced the production of cytokines and chemokines (Figure 6A,B). Upon stimulation with LPS, LDN secreted significantly more IL‐1β, IL‐6, MCP‐1, TNF, G‐CSF and granulocyte–macrophage colony‐stimulating factor compared to NDN (Figure 6A), but not IL‐8, MIP1α and MIP1β, indicating a degree of selectivity (Figure 6B). To ensure that the increase in cytokine production in LDN was not simply due to differential viability (Figure 5A), cytokine production was assessed using ELISpot, which maps cytokine release to individual cells. We focussed on IL‐6 as this cytokine is important in SLE^38^ and neutrophils from healthy donors do not produce IL‐6 in response to LPS.^39^ IL‐6 production was measured at 6 h, when there were minimal differences in viability between neutrophil subsets, and again after 16 h. Similar to healthy controls, NDN from GD secreted little IL‐6 in response to LPS at 6 or 16 h. In contrast, LDN produced more IL‐6 as measured by both the number of spots and the amount of IL‐6 released from each cell (secretory activity, Figure 6C–E). Importantly, increased IL‐6 production was evident at 6 h when there was little difference in cell viability between neutrophil subsets, demonstrating that LDN have greater capacity to produce proinflammatory cytokines (Figure 6A).

### 3.11 CD98^+^ LDN in SLE are functionally equivalent to CD98^+^ LDN from GD

**FIGURE 7:**
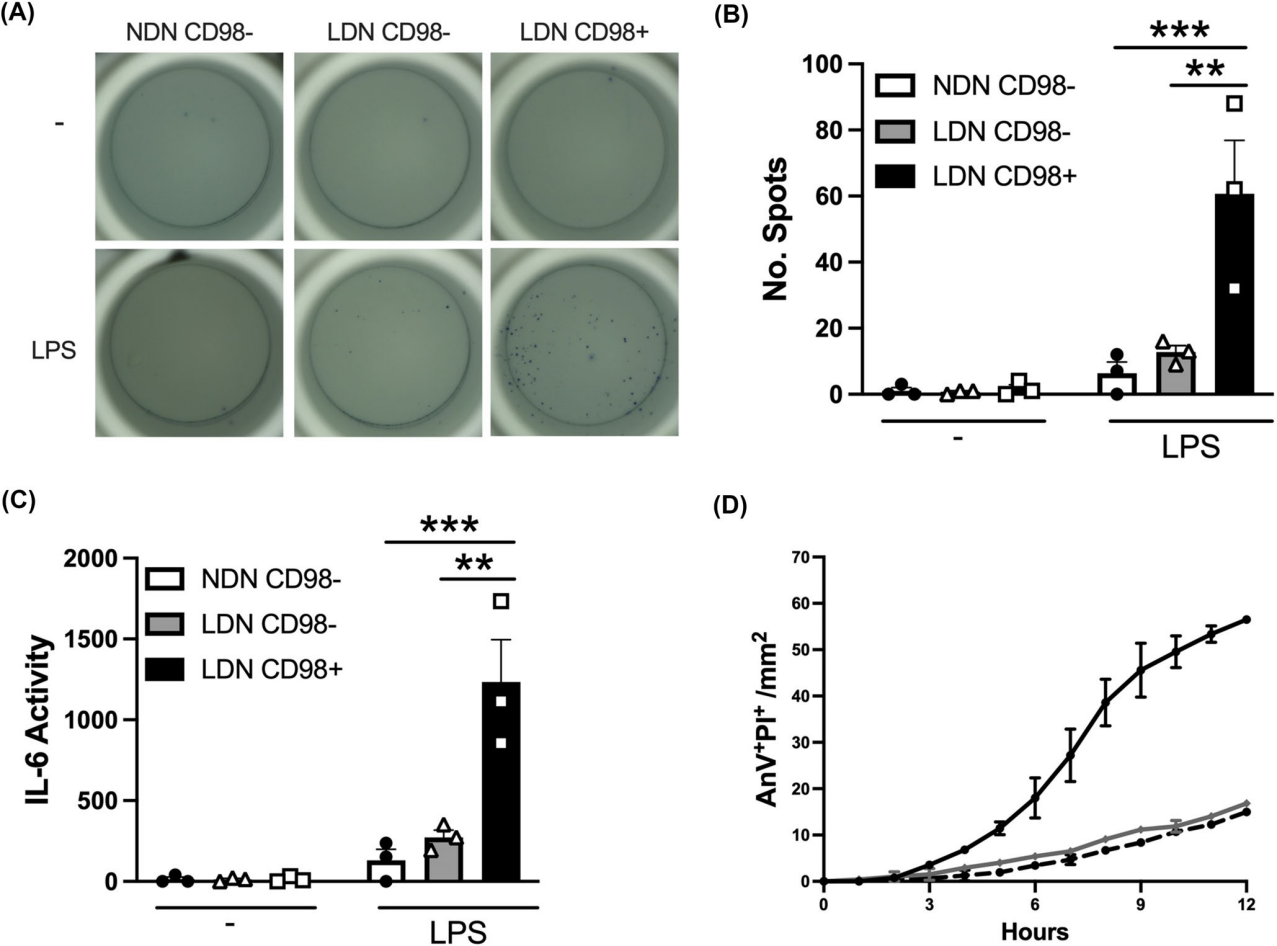
CD98^+^ low‐density neutrophils (LDN) in systemic lupus erythematosus (SLE) are functionally similar to CD98^+^ LDN from healthy donors treated with granulocyte colony‐stimulating factor (G‐CSF) (GD). (A) Representative images of lipopolysaccharides (LPS)‐induced interleukin‐6 (IL‐6) secretion using ELISpot assays. Quantification of (B) the number of cells secreting IL‐6 and (C) IL‐6 secretory activity (*n* = 3). (D) Spontaneous apoptosis of CD98^−^ normal‐density neutrophils (NDN) (black), CD98^−^ LDN (grey) and CD98^+^ LDN (dashed) was measured over 13 h with the IncuCyte S3 System 9 (*n* = 2). Data are mean ± standard error of mean (SEM). ^**^ *p* < .01, ^***^ *p* < .001, two‐way analysis of variance (ANOVA)

Enriched populations of CD98^−^ NDN, CD98^−^ LDN and CD98^+^ LDN from the PB of SLE patients were isolated and the ability to produce IL‐6 in response to LPS was assessed using ELISpot (Figure 7A–C). Consistent with GD, CD98^−^ NDN did not produce IL‐6 in response to LPS stimulation. Similar results were observed with CD98^−^ LDN, with only a small number of cells secreting IL‐6 after LPS stimulation. Importantly, CD98^+^ LDN produced more IL‐6 as measured by the number of spots (Figure 7B) and activity (Figure 7C) compared with CD98^−^ NDN or LDN. These findings indicate that CD98^+^ LDN in SLE donors are functionally equivalent to the LDN population in GD. We also assessed the ability of these three neutrophil subsets to undergo spontaneous apoptosis. In line with results from the GD, CD98^−^ NDN underwent spontaneous apoptosis when placed in culture. In contrast, both CD98^−^ LDN and CD98^+^ LDN were relatively resistant to apoptosis, with few cells dying after 12 h.

## 4 DISCUSSION

In this study, we took advantage of the finding that G‐CSF‐mobilises LDN from healthy donors^11^, ^26^ and performed proteomic analysis to characterise this population in more detail than previously possible. Our results reveal that LDN from GD are a relatively long lived population of neutrophils with an enhanced capacity to produce proinflammatory cytokines. Furthermore, LDN have enhanced metabolic flexibility as a result of the ability to engage both glycolysis and mitochondrial‐dependent pathways to produce ATP. Importantly, as LDN are thought to play pathogenic roles in SLE and rheumatoid arthritis,^6^, ^7^, ^11^, ^26^ results from our study highlight key mechanisms responsible for their ability to drive inflammation and tissue damage in these autoimmune diseases.

Previous reports have shown that the appearance of LDN in PB can correlate with disease activity, inflammation and tissue damage in SLE.^5^, ^14^, ^29^ As such, identification of LDN in PB could provide a biomarker for disease activity or response to treatment. This approach is currently limited by the lack of phenotypic markers for LDN and the need for density‐gradient separation. We found that CD98 expression was able to identify LDN in PB of SLE patients without density‐gradient separation. CD98 is an essential amino acid transporter mediating the uptake of branched chain and aromatic amino acids. Transport of amino acids via CD98 can fuel cellular metabolism via OXPHOS and activate the mTOR signalling pathway.^28^ While typically expressed at low levels on normal neutrophils,^28^ LDN from GD displayed high levels of CD98 compared to NDN, with levels equivalent to those observed on monocytes. Monocytes and macrophages strongly express CD98, where it may play a role in cellular differentiation and can facilitate IL‐1β and TNFα production.^40^ In a small cohort of SLE patients, the number of CD98^+^ neutrophils in PB appeared to correlate with disease activity. CD98^+^ neutrophils may therefore represent a novel prognostic biomarker. Consistent with our observations on G‐CSF mobilised LDN, CD98^+^ neutrophils from SLE patients have a low‐density phenotype and comprise both mature and immature cells. Whether CD98^+^ LDN are derived from a population in the bone marrow (BM) that is mobilised to PB in response to G‐CSF, or if G‐CSF upregulates CD98 on LDN remains to be determined. Importantly, CD98^+^ LDN from SLE patients were functionally equivalent to those isolated from GD, with an enhanced capacity to produce IL‐6 and increased resistance to apoptosis.

LDN are often considered to be a distinct subset of neutrophils; however, our findings indicate that there is further heterogeneity within this population. While all GD LDN expressed high levels of CD98, not all LDN from SLE patients expressed CD98. Functionally, CD98^+^ LDN from SLE have the same enhanced capacity to produce IL‐6 and resistance to apoptosis as GD LDN. Heterogeneity within the LDN has been previously noted. A study on LDN isolated from healthy individuals demonstrated functional similarity to NDN, with both subsets producing similar levels of ROS and undergoing cell death via apoptosis.^41^ More recently, single‐cell transcriptional profiling of neutrophils from healthy donors treated with G‐CSF or patients with pancreatic cancer found that LDN are heterogenous, with neutrophils at all stages of differentiation.^26^ Taken together, our data support the concept that neutrophil density alone is not sufficient to identify a discrete population of neutrophils, but rather that function of LDN may depend on the disease or inflammatory environment.^41^ Future research will focus on characterising functional differences between CD98^+^ and CD98^−^ LDN and understanding how this contributes to the pathogenesis of SLE.

We revealed an enrichment of proteins and metabolites associated with mitochondrial respiration, which translates to increased energetic capacity of LDN compared to NDN. Conventional neutrophils rely primarily on glucose and glycolysis to meet energy demands, with little reliance on mitochondrial ATP production.^2^, ^42^ In nutrient‐depleted environments, such as within inflamed tissue, NDN may have less ability to maintain energy demanding functions, such as generation of ROS or NETosis.^43^, ^44^ We confirmed that NDN are highly glycolytic and produce little ATP through OXPHOS, even in the absence of glucose.^45^ In contrast, G‐CSF mobilised LDN were able to produce ATP through both glycolytic and mitochondrial‐dependent pathways. Low glucose environments could significantly reduce ATP production and the ability to maintain metabolically demanding functions in NDN, but this would be less of a limitation for LDN because of greater metabolic flexibility. Our study strongly suggests that human LDN are more adaptable to metabolically demanding, nutrient‐depleted inflammatory microenvironments. Other evidence to support this contention comes from a study of LDN in a murine model of breast cancer. In contrast to NDN, immature LDN continued to generate NETs and ROS in the absence of glucose by relying on the uptake of glutamate and proline to support mitochondrial‐dependent metabolism.^10^, ^43^ The ability to form NETs within the tumour microenvironment in turn supported tumour growth and the formation of liver metastases.^10^ Our study also found increased activation of the mTOR signalling pathway, which may facilitate increased production of NETs by LDN.^46^ Given that the immune microenvironment in SLE can promote the metabolic reprogramming of other immune cell populations to enhance pathogenic capacity,^47^ we propose that CD98 contributes to the metabolic flexibility of LDN.

In keeping with the known role for CD98 in essential amino acid transport,^27^, ^48^ we found increased intracellular levels of essential amino acids in LDN. Significantly, inhibition of CD98 with JPH203 completely blocked the metabolic flexibility of LDN but had no effect on NDN. This result demonstrates that CD98‐mediated amino acid uptake is required to fuel the metabolic activity of LDN. Our results align with those from a mouse tumour model, where immature LDN were able to sustain metabolically demanding functions in a nutrient‐depleted environment by utilising amino acids as an alternative fuel source. Deprivation of these amino acids impaired energy production and the ability of immature LDN to form NETs.^10^ Non‐essential amino acids glutamine and proline were identified as the dominant fuel sources used by immature murine LDN; however, our data indicate that human LDN may preferentially use essential amino acids rather than glutamine and proline as an alternate fuel source. Our ongoing studies are focussed on understanding how targeting CD98 and limiting amino acid updates affect the function of LDN in vivo.

CD98‐mediated amino acid uptake is also known to activate the mTOR signalling, and in line with this, LDN had an increase in proteins associated with this pathway. In the context of neutrophils, mTOR activation regulates mitochondrial metabolism, NET formation,^49^ modulates neutrophil chemotaxis^31^ and inhibits autophagy.^46^ Increased activation of this pathway may explain the enhanced ability of LDN to generate NETs and could regulate their migration from the blood into inflamed tissues.^6^, ^12^ It is possible that targeting CD98‐mediated amino acid uptake with JPH203^33^ or inhibition of the mTOR signalling pathway^31^ may limit the pathogenic capacity of LDN in SLE. To address this question directly, our future research will focus on specific deletion of CD98 from neutrophils in relevant murine models of SLE. This approach will explore how the CD98 neutrophil population contributes to disease in vivo and whether targeting CD98 can limit tissue damage.

Previous studies on the LDN subset have focussed on mice.^10^ While a similar increase in mitochondrial energy production was observed in murine LDN, there are species differences. Murine LDN are able to engage mitochondrial respiration in the absence of glucose, which was rapidly reversed following the addition of glucose, suggesting that murine LDN are geared towards glycolysis.^10^ In contrast, human LDN increased mitochondrial respiration in the absence of glucose, and this was maintained even after the addition of glucose, indicating that human LDN produces ATP through glycolysis and OXPHOS, irrespective of access to glucose. Surprisingly, human LDN had significantly less mitochondrial mass compared to both mature and immature NDN. This was unexpected given the increased mitochondrial respiration observed in human LDN and previous reports that murine LDN have higher mitochondrial mass.^43^ Despite this, LDN had similar mitochondrial membrane potential, suggesting that the mitochondria in human LDN are more active.

This study also identified other functional differences between LDN and NDN that may contribute to the pathology of SLE. We found that LDN could secrete more cytokines and chemokines than NDN. In fact, LDN generated over 40‐fold more IL‐6 in response to LPS. This enhanced ability to generate IL‐6 may be particularly important in SLE as it induces the terminal differentiation of B cells into antibody‐producing plasma cells. IL‐6 inhibition reduced anti‐dsDNA autoantibodies, renal damage and mortality in an animal model of SLE.^50^ LDN in SLE have been associated with the type I IFN signature, including an enhanced capacity to synthesise IFNα as well as increased abundance of type I IFN‐regulated proteins compared to NDN.^5^, ^23^ However, in our proteomic analysis, we found no evidence for a type 1 IFN signature in LDN isolated from GD. Production of IFNα was undetectable in isolated NDN and LDN at baseline or following stimulation with LPS (data not shown). We speculate that G‐CSF exposure alone is insufficient to prime LDN to produce type I IFNs or that LDN might drive IFN production by other cell types.

The lifespan of a neutrophil is tightly regulated. While extravascular survival is necessary to ensure that pathogens are effectively eliminated, neutrophils readily undergo apoptosis to prevent tissue damage.^51^ We therefore explored the susceptibility of LDN and NDN to cell death. We found that LDN had delayed spontaneous apoptosis and survived longer than NDN.^7^, ^52^ Our results suggest that IAP proteins are likely responsible for this enhanced survival as treatment of LDN with Smac‐mimetics increased apoptosis and restored susceptibility to cell death to levels comparable with NDN.^35^ While normal neutrophils mainly express X‐linked IAP (XIAP), cellular IAP2 (cIAP2) is known to be selectively upregulated by G‐CSF.^53^ Therefore, it is possible that the resistance to apoptosis observed in LDN is driven by increased expression of these proteins, or that XIAP or cIAP3 are upregulated in response to G‐CSF. While LDN were relatively resistant to all forms of apoptosis, they died rapidly by necroptosis. Necroptosis is a lytic form of cell death that results in release of neutrophil‐derived cytotoxic proteins and proinflammatory mediators.^36^ We therefore suggest that LDN are geared toward lytic cell death, which may also contribute to tissue damage in SLE.

## 5 CONCLUSION

We report that CD98 provides a phenotypic marker for LDN. CD98 is a transporter of essential amino acids with low‐level expression on NDN. CD98^+^ LDN produce ATP through both glycolytic and mitochondrial‐dependent pathways, allowing flexibility for metabolically demanding functions, such as the generation of ROS or NETs. Expression of CD98 is also associated with activation of mTOR pathway. Furthermore, LDN are relatively resistant to apoptosis and can survive longer than NDN. However, LDN retain susceptibility to necroptosis, which may contribute to their proinflammatory potential. Inhibition of CD98 with a molecule currently in clinical trials for cancer (JPH203) reduced the metabolic flexibility of LDN. CD98 inhibition could therefore provide a novel therapeutic approach to diseases associated with LDN, including SLE.

## CONFLICTS OF INTEREST

Laboratory of Ian P. Wicks has received funding from CSL Pty Ltd. for research on antagonists of haemopoietic growth factors. Nicholas Wilson is employed by CSL Pty Ltd.

## Supporting information

Supporting Information

Supporting Information

Supporting Information

Supporting Information
