# Supplementary material for: CD98 defines a metabolically flexible, proinflammatory subset of low‐density neutrophils in systemic lupus erythematosus
Source: Clin Transl Med. 2023 Jan 18;13(1):e1150. doi: 10.1002/ctm2.1150 (PMC9849148; doi:10.1002/ctm2.1150)
Supplement: Supplementary file 1 — Supporting Information [file CTM2-13-e1150-s002.docx]

**
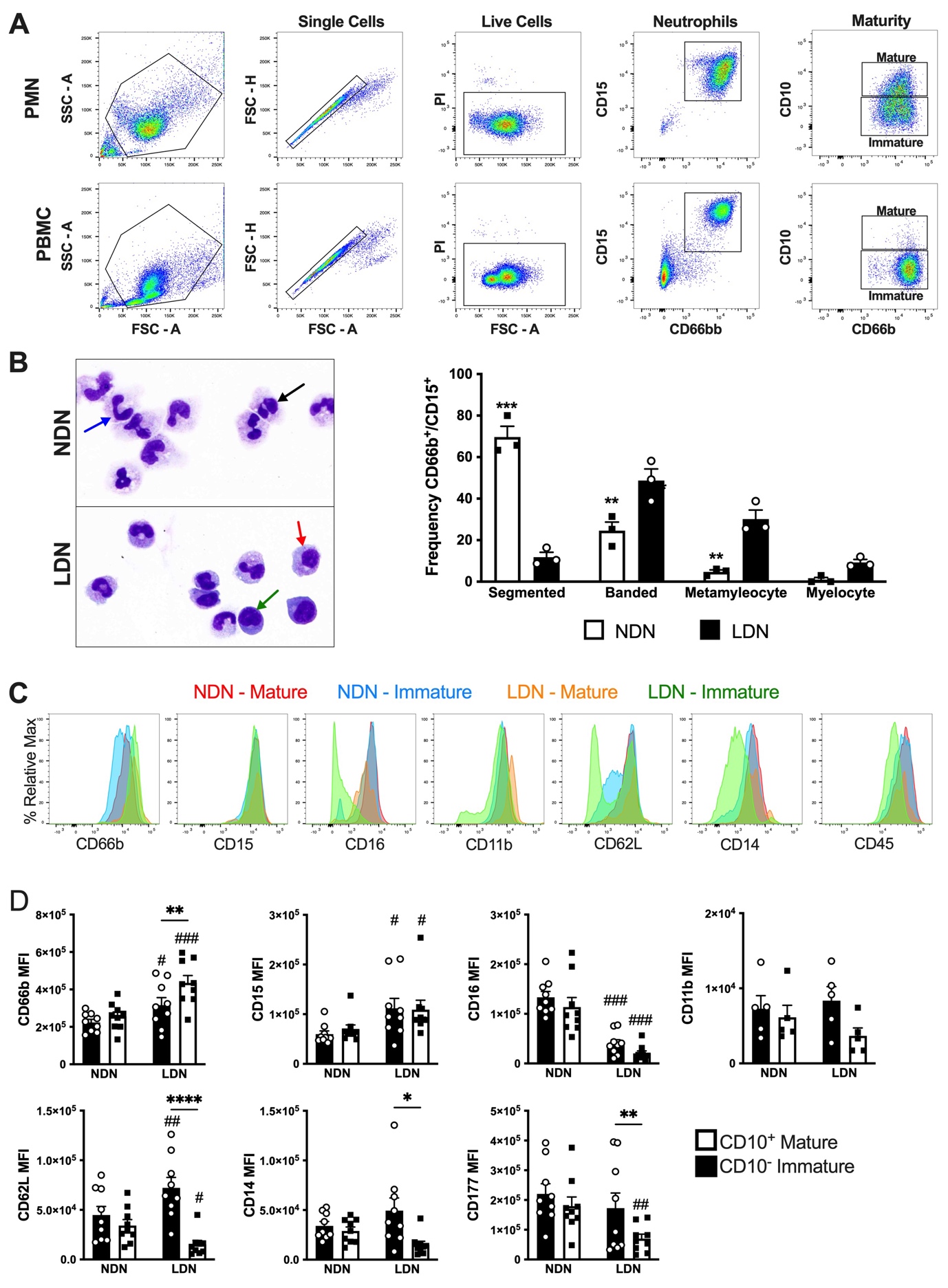
**

**Sup Fig 1 G-CSF administration mobilizes a population of LDN into peripheral blood.** (**A**) Representative dot blots of the gating strategy used to identify neutrophils in the PMN and PBMC layers after density gradient separation. (**B**) Representative images from May-Grünwald Giemsa staining of NDN (white) and LDN (black) and the frequency of segmented neutrophils (black arrow), band neutrophils (blue arrow), metamyelocytes (green arrow) and myelocytes (red arrow) in each neutrophil layer (n=3). (**C**) Expression of neutrophil maturation and activation markers represented as histograms of the MFI (**D**) Levels of CD66b, CD15, CD16, CD11b, CD14 and CD62L on CD10^+^ (black) and CD10^-^ (white) NDN and LDN from GD, represented as MFI (n=9 except CD11b where n=5). Data are mean ± SEM. ^#/^* p < 0.05, ^##/^** p < 0.01, ^###/^*** p < 0.001, Student t-test or one-way ANOVA.

**
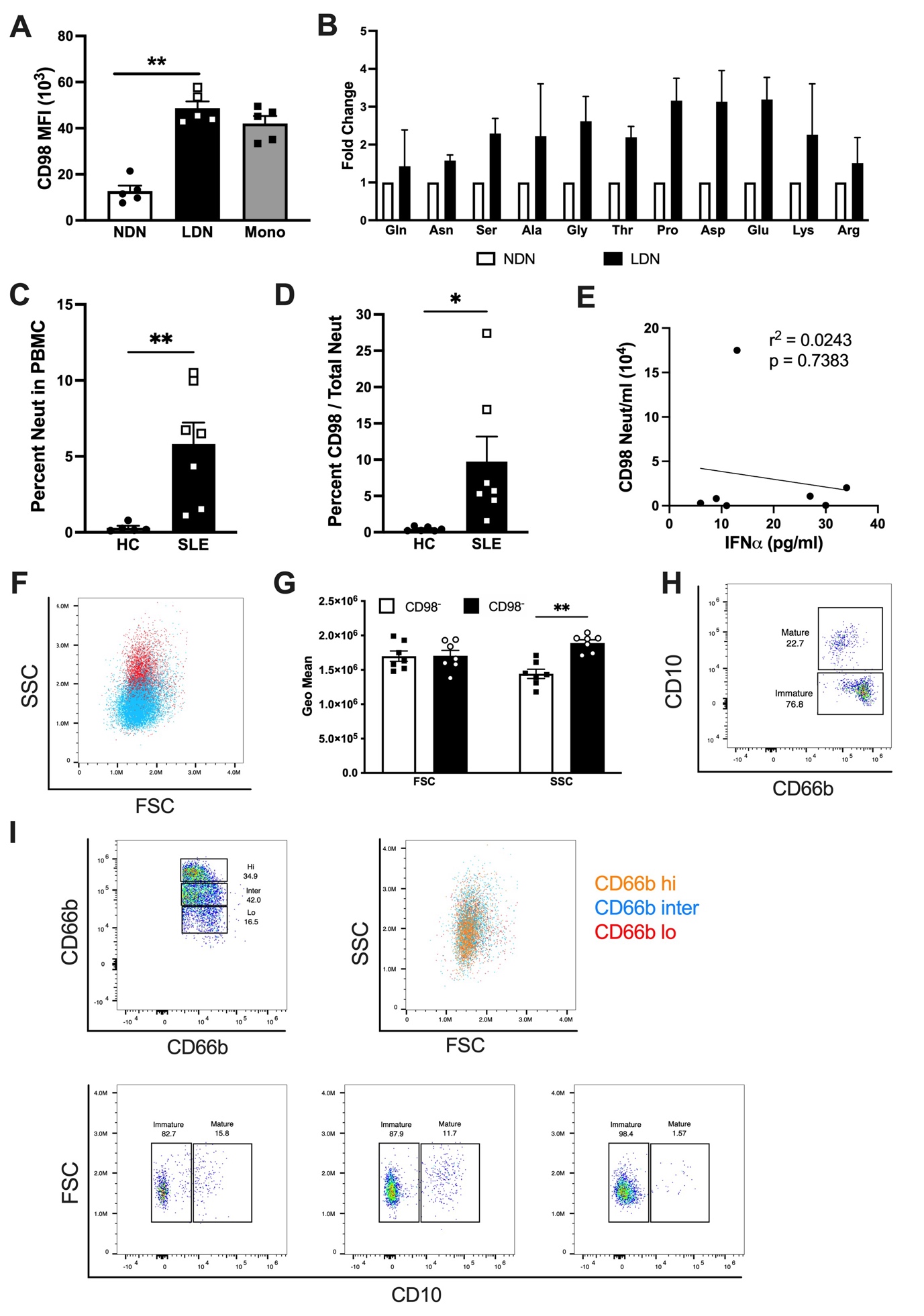
**

**Sup Fig 2 CD98^+^ neutrophils are found in SLE.** (**A**) Membrane expression of CD98 on NDN (white), LDN (black) and monocytes (grey) quantified by MFI (n=5). (**B**) Fold change of intracellular levels of essential amino acid content in LDN relative to NDN (n=5). (**C**) Proportion of CD66b^+^/CD15^+^ neutrophils present within the PBMC layer after density separation of PB from HC (n=6) and SLE patients (n=7). (**D**) Percent of CD98^+^ neutrophils in the total neutrophil population in the PB of healthy controls (n=6) and SLE patients (n=7). (**E**) Correlation between CD98^+^ neutrophils in PB and levels of IFNα in patient plasma (n=7). (**F**) Representative dot blot showing forward (FSC, size) and side (SSC, granularity) scatter of CD98^+^ and CD98^-^ neutrophils (n=7). (**G**) Mean FSC and SSC of CD98^+^ and CD98^-^ neutrophils (n=7). (**H**) Representative dot blots showing mature and immature CD98^+^ LDN in the PB of a patient with active SLE (n=7). (**I**) Representative dot blots characterizing the size, granularity, and maturation of CD98^+^ neutrophils with high, intermediate, and low CD66b expression levels (n=7). Data are mean ± SEM. *p < 0.05, **p < 0.01***, p < 0.001, Students t-test or one-way ANOVA.

**
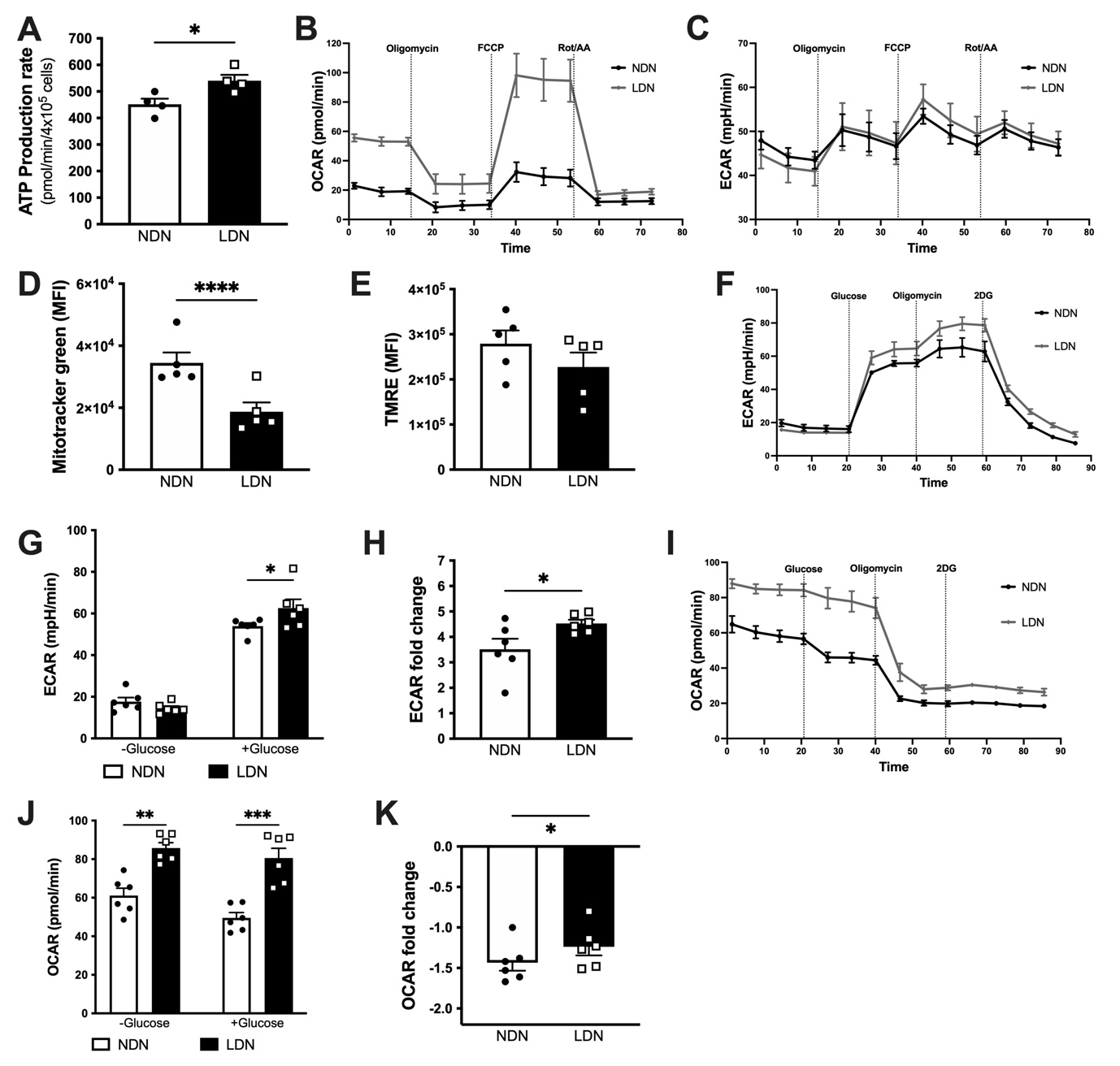
Sup Fig 3 Assessment of cellular metabolism in CD98^-^ NDN and CD98^+^ LDN.** (**A**) Total ATP production rate in NDN (white) and LDN (black) from GD (n=4). (**B**) OCAR and (**C**) ECAR levels observed during the mitochondrial stress test (oligomycin 1.26 µM, Carbonyl cyanide-4-(trifluoromethoxy) phenylhydrazone (FCCP) 660 nM, Rotenone 100 nM and antimycin A 1 µM) (n=4). (**D**) MFI of MitoTracker™ Green and (**E**) TMRE staining of both neutrophil subsets (n=5). (**F**) ECAR levels obtained during the glycolysis stress test (10 µM glucose, oligomycin 1 µM and 50 mM 2DG, n=6). (**G**) Absolute ECAR values and (**H**) ECAR fold change in ECAR in neutrophil subsets following the addition of 10 mM glucose (n=6). (**I**) OCAR levels obtained during the glycolysis stress test was performed on both neutrophil subsets (n=6). (**J**) Absolute OCAR values and (**K**) fold change in OCAR in NDN and LDN following the addition of 10 mM glucose (n=6). Data are mean ± SEM. *p < 0.05, **p < 0.01***p < 0.001, one-way ANOVA.

**
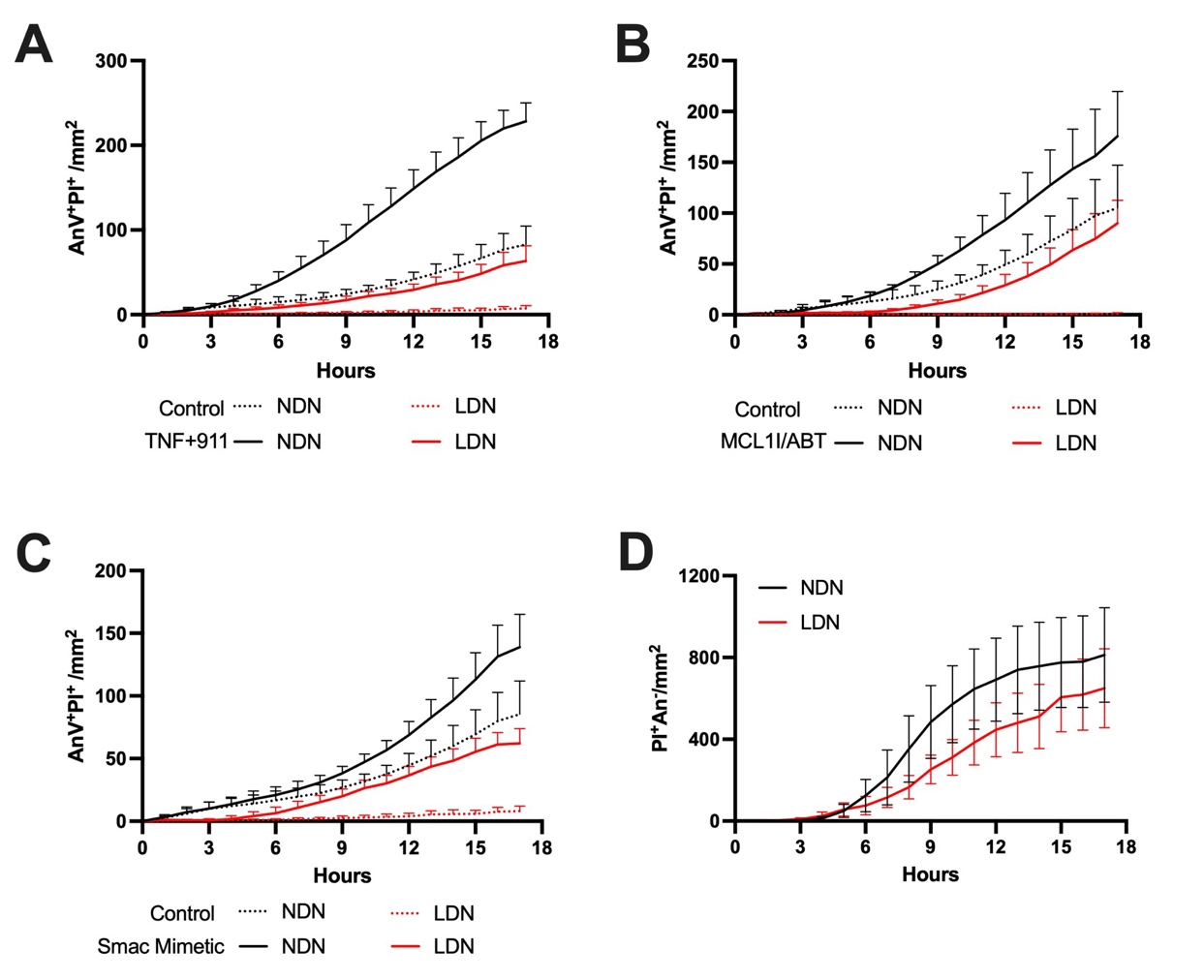
**

**Sup Fig 4 Kinetics of neutrophil death.** (**A**) Extrinsic and (**B**) intrinsic apoptosis stimulated by TNA and Smac-mimetic or ABT-737 and Mcl-1 inhibitor, respectively. NDN are represented in black and LDN in red. Death was measured using the Incucyte, and plates were imaged every hour for 18h (n=3-5). (**C**) Neutrophils were treated with Smac-mimetic and cell death measured as above (n=5). (**D**) Neutrophil were treated with a necroptotic stimulus (TSI). Cell death was measured using the IncuCyte and appearance of AnV^-^/PI^+^ cells over 18 hours (n=8). Data are mean ± SEM. *p < 0.05, **p < 0.01***, p < 0.001, one-way ANOVA.
